# Supplementary material for: Robust machine learning segmentation for large-scale analysis of heterogeneous clinical brain MRI datasets
Source: Proc Natl Acad Sci U S A. 2023 Feb 21;120(9):e2216399120. doi: 10.1073/pnas.2216399120 (PMC9992854; doi:10.1073/pnas.2216399120)
Supplement: Supplementary file 1 — Appendix 01 (PDF) [file pnas.2216399120.sapp.pdf]

Robust machine learning segmentation for large-scale analysis of heterogeneous clinical brain MRI datasets – Supplementary materials

Benjamin Billot, Colin Magdamo, You Cheng, Steven E. Arnold, Sudeshna Das, Juan Eugenio Iglesias

Supplement 1: Demographic information and imaging protocols for the large clinical cohort.

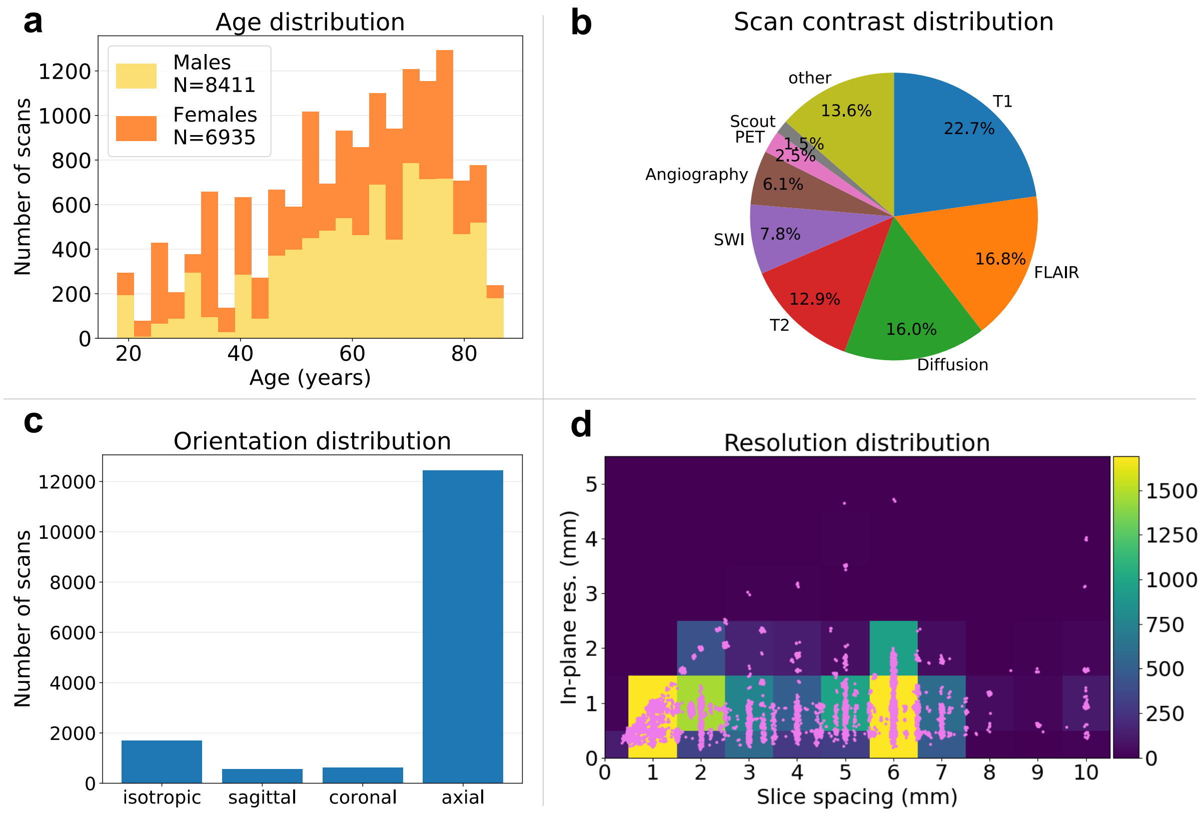

Fig. S1. Demographic information and imaging protocols for the 15,346 clinical scans acquired during 1,367 subject sessions at the Massachusetts General Hospital. (a) Age and gender of the subjects, given per scan. (b) Distribution of the used contrast. SWI refers to sensibility weighted imaging, PET to positron emission tomography, and "other" to either unidentified contrasts (9.7%) or sequences with sub-percentage occurrence (e.g., gradient-echo, contrast-agent). (c) Distribution of the acquisition direction (i.e., scan orientation). (d) Distribution of the scan resolution. Individual scans are represented by pink dots over the underlying density distribution.

Supplement 2: Examples of erroneous segmentations by *SynthSeg*$^+$

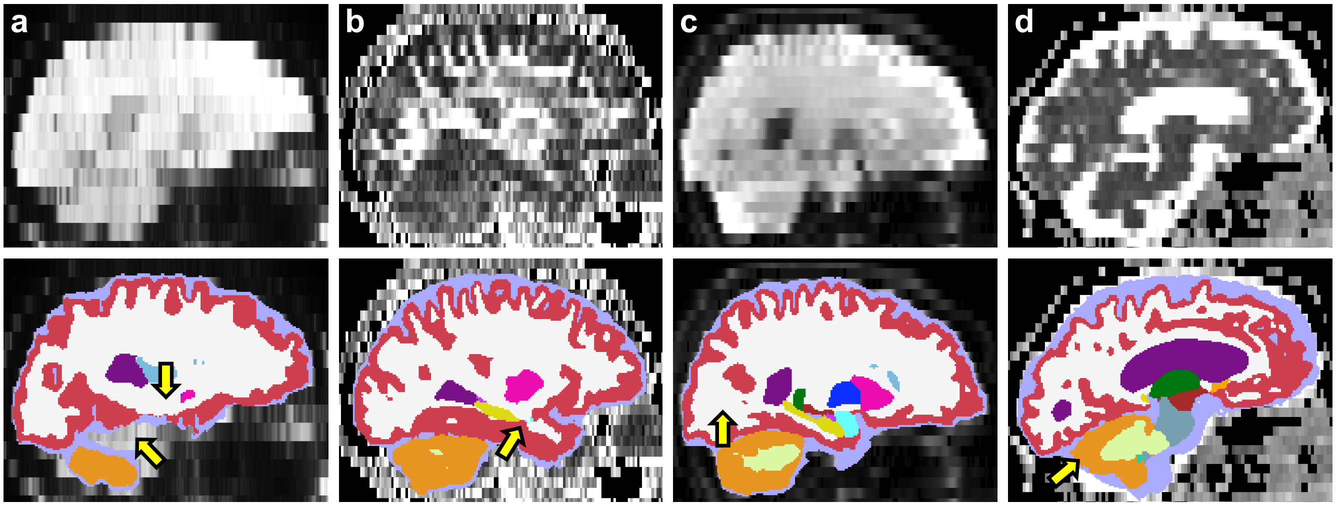

Fig. S2. Representative samples of failed segmentations obtained by visual QC. We highlight that these images are very challenging to segment either because of low tissue contrast, low signal-to-noise ratio, or very low resolution. (a) Part of the cerebellum and most subcortical regions are missing. (b) The amygdala is segmented as cerebral cortex (red). (c) Missing posterior lateral ventricle (dark purple), which is instead segmented as white matter (white). (d) The cerebellum is over-segmented in the posterior direction.

Supplement 3: ROC curves for the automated QC methods

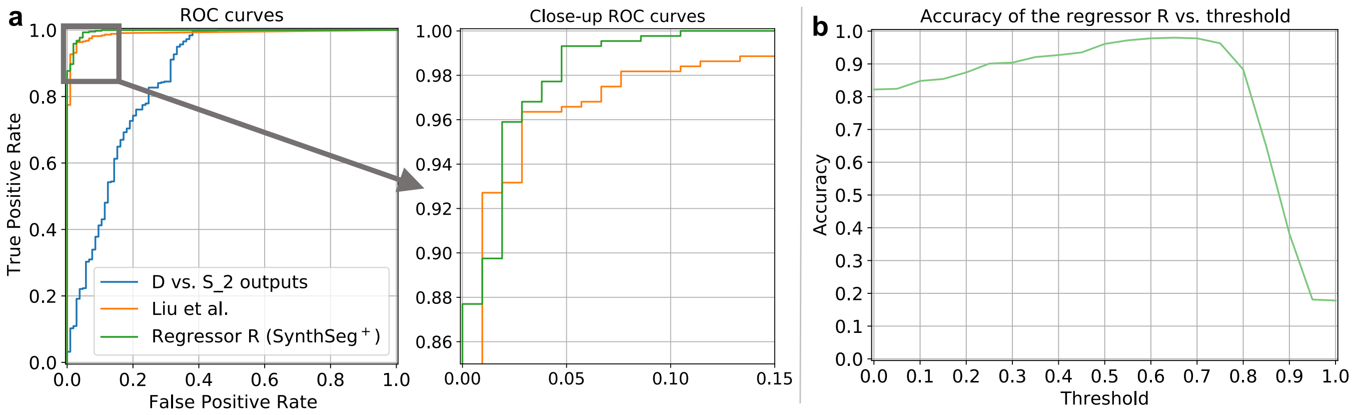

Fig. S3. (a) ROC curves of the three methods for automated quality control analysis, with a close-up on the upper left corner. They show that the denoiser-based strategy (i.e., D vs. S_2) performs the worst. The state-of-the-art method proposed by Liu et al. obtains much better results, but it very slightly outperformed by the regression-based technique implemented in *SynthSeg*$^+$, although no statistical difference is found between the two.(b) Accuracy of our regression-based method as a function of the threshold. Our method is relatively robust to the chosen value, as the accuracy only varies by 0.01 for thresholds between 0.55 and 0.75.

Supplement 4: Quantitative evaluation of the robustness to different subject populations

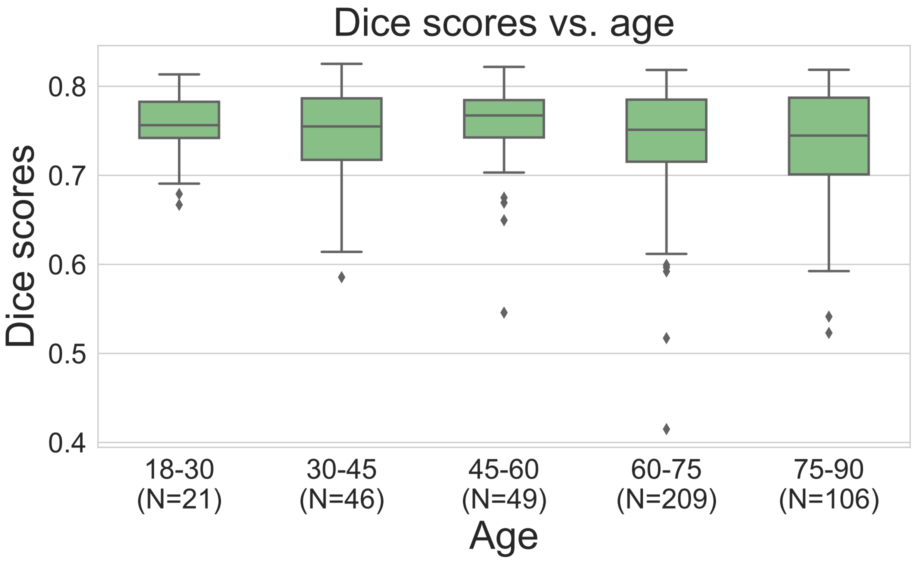

Fig. S4. Here we study the accuracy of *SynthSeg*$^{+}$ as a function of age, which we take as a proxy for morphological variability. We plot the Dice scores obtained by the proposed segmentation algorithm on the 500 clinical scans with ground truth. The results show that *SynthSeg*$^{+}$ maintains a high level of accuracy across the whole age range. This is further highlighted by the absence of statistical difference at the p=0.05 level when computing two-sided Wilcoxon signed-rank tests to compare each group with its neighbouring class to the left (minimum p-value of 0.062 between 45-60 and 60-75).
